# Assessing Trauma Center Accessibility for Healthcare Equity Using an Anti-Covering Approach

**DOI:** 10.3390/ijerph19031459

**Published:** 2022-01-27

**Authors:** Heewon Chea, Hyun Kim, Shih-Lung Shaw, Yongwan Chun

**Affiliations:** 1 Department of Geography, University of Tennessee at Knoxville, 1000 Phillip Fulmer Way, Knoxville, TN 37996-0925, USA; hkim56@utk.edu (H.K.); sshaw@utk.edu (S.-L.S.); 2 Geospatial Information Sciences, University of Texas at Dallas, 800 W. Campbell Rd., Richardson, TX 75080-3021, USA; ywchun@utdallas.edu

**Keywords:** trauma center, accessibility, anti-covering location problem, ACLP, Getis–Ord G, vehicle accident

## Abstract

Motor vehicle accidents are one of the most prevalent causes of traumatic injury in patients needing transport to a trauma center. Arrival at a trauma center within an hour of the accident increases a patient’s chances of survival and recovery. However, not all vehicle accidents in Tennessee are accessible to a trauma center within an hour by ground transportation. This study uses the anti-covering location problem (ACLP) to assess the current placement of trauma centers and explore optimal placements based on the population distribution and spatial pattern of motor vehicle accidents in 2015 through 2019 in Tennessee. The ACLP models seek to offer a method of exploring feasible scenarios for locating trauma centers that intend to provide accessibility to patients in underserved areas who suffer trauma as a result of vehicle accidents. The proposed ACLP approach also seeks to adjust the locations of trauma centers to reduce areas with excessive service coverage while improving coverage for less accessible areas of demand. In this study, three models are prescribed for finding optimal locations for trauma centers: (a) TraCt: ACLP model with a geometric approach and weighted models of population, fatalities, and spatial fatality clusters of vehicle accidents; (b) TraCt-ESC: an extended ACLP model mitigating excessive service supply among trauma center candidates, while expanding services to less served areas for more beneficiaries using fewer facilities; and (c) TraCt-ESCr: another extended ACLP model exploring the optimal location of additional trauma centers.

## 1. Introduction

Geographical accessibility to health care facilities is essential to ensure safer and healthier lives. The more convenient and the better the access to healthcare facilities, the more beneficial they are in meeting patients’ medical needs. Depending on the types of medical needs, different levels of accessibility to medical services are available. Of concern in this research, trauma centers are specialized medical facilities designed to cope with unexpected accidents or illnesses requiring the most time-sensitive healthcare [1,2,3,4]. To receive timely and appropriate treatment, a patient must reach to a trauma center as soon as possible. A delay in getting to medical experts may result in long-term effects or death [5,6,7]. Depending on the type of accident or illness, the time patients have to receive appropriate treatment to survive without post-disorders differs. For example, certain conditions, such as heart attack or cerebral hemorrhage, must be treated by a specialist within one hour in order for patients to recover without side effects [8,9,10,11]. The faster the response to patients with such symptoms as severe bleeding, complex fractures, and amputations, the lower the likelihood of death or disability [12,13]. When a traumatic situation occurs, the patient’s arrival to the hospital within an hour, called the “golden hour,” increases the chances of survival and recovery [14,15]. This is a pervasive issue around the world.

In Tennessee, motor vehicle accidents are among the most prevalent causes of traumatic injury in patients needing transport to a trauma center, and 8674 cases in Tennessee are categorized as trauma accidents resulting from motor vehicle crashes in 2018 [16]. However, trauma centers are not evenly located for best serving potential demand. Instead, there are extreme geographic disparities in medical service access with trauma centers generally clustered in urban areas and virtually absent in rural areas [1]. Consequently, not all vehicle accidents occur within an hour’s travel time distance by ground transportation to the nearest trauma center. This phenomenon raises issues regarding the equity of medical service [17]. Therefore, assessments measuring this discrepancy and identifying ideal locations for trauma centers are critical.

From a planning perspective, resolving the spatial disparity in the access to trauma centers is directly associated with socioeconomic equity and public spending. Efforts have been made via optimization modeling and solution methods to identify optimal locations and to assign demands [18]. This study has four goals. First, we assess the difference in accessibility to trauma centers by focusing on the locations of motor vehicle accidents in Tennessee and the resulting fatalities. Second, we present a method for relieving the spatial disparity in accessibility to trauma centers based on identified clusters of underserved severe vehicle accidents by using the anti-covering location problem (ACLP) and a spatial association measure, Getis–Ord’s G. The ACLP is a coverage-based dispersion model. Giving this model its name, Moon and Chaudhry [19] first attempted to distinguish it from other prominent covering models. According to Church and Murray [20], “The anti-cover location problem seeks to maximize the total weighted benefit of facilities sited in a region, doing so in a manner that ensures at least a minimum pre-specified distance or travel time between facilities and demand is maintained.” ACLP is useful in planning—for example, locating nuclear facilities a certain distance from residential areas and/or other nuclear facilities. Depending on the type of facility and the purpose, ACLP has many variations. Third, the excessive supply of trauma centers for each demand is treated in moderate numbers to improve the ACLP model’s results. This part may contribute to clarify how many centers are moderate numbers of trauma center from potential demands. Fourth, the ACLP model identifies the best locations for additional trauma centers in Tennessee.

This study’s analysis consists of four parts. The first part measures service coverage areas based on the diverse cost of travel time to existing trauma centers and presents the discrepancies between accessibility to trauma centers and demands. It is essential to investigate how many demand points are currently covered by Tennessee’s trauma centers based on different limited travel times and how effectively those trauma centers are distributed in order to delineate commonly covered service areas. The second part uses the ACLP models to identify optimal locations for trauma centers while maximizing the number of beneficiaries. The third part addresses the issue of excessive service areas geographically served by multiple trauma centers; those areas are referred to as commonly covered service areas (CSAs). The fourth part examines an extended model offering the optimal solution for identifying additional trauma center locations to supplement Tennessee’s existing trauma centers as of 2019.

## 2. Background

### 2.1. Inequality in Accessibility to Healthcare Services

Emergency healthcare services (EMS) are time dependent. Many emergency care patients are in a life-or-death situation for which only timely assistance can increase the chance of survival and recovery. Trauma care is the most time-dependent service among EMS. Certain locations are frequently sites of critical accidents or emergency medical situations, but the patients may not have access to vital services when they face a life-or-death situation at a location far away from emergency healthcare facilities. Hashmi et al. [6] explored the quantitative state-level relationship among the proportion of pre-hospital deaths, age-adjusted mortality, and timely access to trauma centers. Poor access to trauma centers is strongly related to pre-hospital death.

The mismatch between the distribution of trauma centers and the population of demand areas with a high probability of accidents results in a spatial disparity in access to trauma centers. While most healthcare services offer a broad range of treatment for general and routine medical issues, trauma centers need specialized medical staff and facilities to carry out urgent care and precise surgeries. An extensive amount of financial and professional human resources is necessary to establish and maintain a trauma center, and there are practical restrictions in securing and operating trauma medical service facilities than any other medical services. Trauma centers are usually located in or adjacent to urban areas because the urban area has better conditions to be qualified with more financial and human resources compared to the rural areas. Therefore, rural accident clusters lack accessibility to adequate healthcare providers, especially at trauma centers. Branas et al. [1] estimate the percentage of residents who have access to trauma centers earlier than 45 or 60 min. The result shows the obvious difference between urban and rural areas’ accessibility to trauma centers. Approximately, 47 million Americans in rural areas have no access within an hour of a trauma center, whereas about 43 million Americans in urban areas have access to 20 or more trauma centers.

Social conditions have also been considered in exploring the relationship between accessibility to emergency medical services or trauma centers and potential demands. Caldwell et al. [21] examined different levels of exposure to social conditions associated with access to health care. Rural areas are at a disadvantage in terms of access to health care facilities. The residential segregation in rural areas worsens access to usual health care services; however, higher medical needs are being met among African Americans [22]. Using the logistic regression model, [17] identified disparities in access to trauma centers in urban versus rural areas. Significant disparities exist for vulnerable populations defined by financial status and context of urban-rural location. Hsia and Shen [23] revealed that the closure of trauma centers since 2001 has disproportionately burdened vulnerable people, such as African Americans, those in poverty, and people residing in rural areas.

The disparities in access to emergency health care service is epidemic worldwide; as a result, much research has assessed the different levels of accessibility to emergency uninsured medical service facilities in Ghana, Africa [24]; Dhaka, Bangladesh [25]; Slovakia [26]; Slovenia [27]; Bavaria, Germany [28]; Milan, Italy [29]; Melbourne, Australia [30]; and Hanoi, Vietnam [31]. Luo et al. [8] measured the access to EMS in Wuhan, China by exploring the differences between what the literature states and the actual trip time from an EMS station to the accident scene and from the accident scene to the nearest hospital. They found differences in accessibility to EMS in different regions and in different time windows (off-peak and peak hours) to be useful information for evidence-based health care planning.

Using practical road network conditions, Pedigo and Odoi [32] investigated the geographic accessibility to EMS from the locations of strokes and myocardial infractions in East Tennessee. Their results found disparities in accessibility to EMS to be useful information for developing evidence-based health care planning. Busingye et al. [33] investigated changes in geographic access to emergency care for heart attacks and strokes based on a network’s travel time distance in Middle Tennessee for a decade (from 1999 through to 2010). Access to emergency health care has improved, but the accessibility disparity in rural community remains. Furthermore, Golden and Odoi [34] investigated the distribution in EMS transportation time for stroke and MI patients and found prehospital delay measured by travel time exceeded the guidelines in two of Tennessee’s EMS agencies.

Some researchers have attempted to better assess accessibility to emergency medical facilities. Using a gravity-based model, Chen et al. [35] evaluated trauma centers’ spatial accessibility and identified counties in Ohio that were potentially underserved. They found a huge disparity in accessibility with ten times more served counties and four times less served counties than an average county. Developing and validating a model to quantify access to definitive care, Tansley et al. [36] identified people with poor accessibility to care in Canada. According to Wei et al. [37], the hospital-capacity–demand ratio for each trauma center can be applied for further assessment. They conducted a cross-sectional study examining geographic access to trauma centers based on trauma incident locations in 32 U.S. states.

Modeling approaches are designed to solve disparity problems in accessing emergency medical services. Toregas et al. [38] examined the location of emergency facilities as a set covering problem. This was one of the first and standard attempts to locate emergency facilities and has greatly impacted subsequent research. The heuristic method for the *p*-median problem has also been used to find sub-optimal solutions and applied to locating an emergency facility under the limited computational power [39]. Advanced optimal location problem models have also been developed to solve location problems. For example, Murray and Grubesic [40] suggested multiple objectives of generalized spatial optimization models. Ye and Kim [41] proposed a network-based covering location problem (Net-CLP) in two sub models of network-based maximal covering location problem (Net-MCLP) and network-based location set covering problem (Net-LSCP). Most recently, Janosikova et al. [26] introduced a bi-criteria mathematical programing model to identify the optimal location for EMS ambulance stations within a short time limit and average response time compared to the current station distribution.

Based on the literature review, there are some pointers to achieve better accessibility to EMS or trauma centers. First, using the practical distance based on the actual network connection or travel time approach is required in assessing emergency medical facilities. Second, identifying the actual locations of potential demand and candidate facilities filtered down increases the model’s precision in terms of computational performance’s solution and efficiency. Third, the simple and objective method of measuring and comparing accessibility to EMS or trauma centers is required to facilitate the interpretation of the results. Fourth, due to the special attributes of EMS or trauma centers that must cover as much demand as possible while considering the efficiency of facility installation and operation, a novel mathematical model that is different from methods used in prior research must be applied.

### 2.2. Travel Time Distance Constraint and Commonly Covered Service Area

To address the issue of spatial disparity of trauma centers using the mathematical model approach while considering the geographic relationship between demands and supplies, we defined two terms: travel time distance constraint (TDC) and commonly covered service area (CSA).

TDC is the operational concept of the maximum distance (i.e., geographic range of coverage) that medical service agents can travel to reach the location of potential demands within a specific time frame. In other words, the potential patients under TDC can access the nearest trauma center within suggested TDC constraints. In this study, TDC was measured by the travel-time-cost distance for the limited time using ground transportation.

Using helicopters to transport trauma patients is the most time-responsive method. The decision to use air transport is made to access trauma accident locations where ground transportation cannot reach the patients due to inaccessibility conditions, such as the unavailability of road networks [4,42]. However, the air transport mode also has key restrictions for use, such as unfavorable weather conditions, a lack of skilled pilots who operate the helicopter in any challenging situation, and failure to secure open space for landing. For these reasons, ground transportation remains the primary way to transfer trauma patients of motor vehicle accidents to near trauma centers [16]. Thus, we focused on ground transportation to assess the accessibility from the vehicle crash location to the trauma center.

To examine the model’s response to different TDCs, we used five travel-time-cost TDCs—30 to 90 min in 15 min intervals. TDCs are produced from the service provider—i.e., hospitals including the current trauma centers and general hospitals—using Tennessee’s road networks during 2019 in a GIS environment.

CSAs are areas served by multiple facilities using their TDCs. From a geometric point of view, CSAs are overlapping intersect areas among TDCs. From a service-level perspective, the demand within a CSA can have more opportunity for medical service, even in the event of facility failure. However, in other perspectives, excessive medical services may indicate if the medical facilities are clustered to create CSAs, while other areas have little coverage, raising the issue of the spatial disparity of healthcare access.

### 2.3. Spatially Autocorrelated Pattern of Demands

Vehicle accidents are the second-highest cause of traumatic injuries in Tennessee. The pattern of vehicle accidents and/or fatalities is clustered over the study area (see Figure 4). Identifying those accidents’ spatial clusters pinpoints the potentially accident-prone locations, and the information can be modeled as weight in the ACLP. To measure the degree of spatial clusters for fatalities, we use Getis–Ord’s global *G* (hereafter, *G-*statistic for convenience) [43]. *G-*statistic measures the level of fatality clustering within the TDC of each potential or existing trauma center (*Y_k_*). The formula of *G-*statistic is given as the following:

(1) $$G_{Y_{k}}=\frac{\sum_{i=1}^{n}\sum_{j=1}^{n}w_{ij}x_{i}x_{j}}{\sum_{i=1}^{n}\sum_{j=1}^{n}x_{i}x_{j}},\;\forall_{j}\neq i$$

where $x_{i}$ and $x_{j}$ present the attribute value of demand location (i.e., fatality) at tessellated units *i* and *j* in TDC of *Y_k_*. $w_{ij}$ is a spatial weight for *i* and *j*, and it is set as either 0 or 1 based on the adjacency between *i* and *j*. n is the number of demand points in TDC of *Y_k_*. Note that *G-*statistic with a low *p*-value (*p* < 0.05 in our analysis) and a positive *z*-score indicates that the demands’ spatial distribution in TDC of *Y_k_* is clustered and that the higher positive value represents the greater degree of clustering. We use *G*-statistic as a weight in the objective function for two ACLP models (see Figure 5 for the modeling scheme) to prioritize the trauma centers with highly clustered TDCs in finding the optimal locations for trauma centers.

When the *G-*statistic is used, one assumption is that the area in TDC is regarded as homogeneous with an identical degree of the *G-*statistic. It is reasonable to assume that the clusters within a certain TDC have a greater chance for acute treatment within the TDC’s predetermined time.

**Figure 1:**
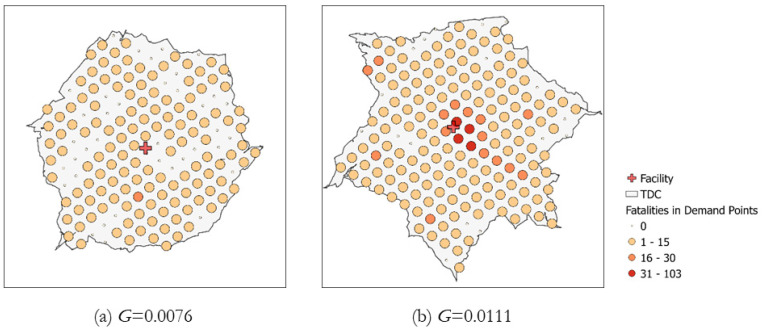
Computations of global *G*-statistic for TDCs with different intensity of fatalities. (**a**) *G* = 0.0076; (b) *G* = 0.0111.

Figure 1 details how *G*-statistic is translated as weight in the ACLP to the geographic pattern of fatalities because the weight represents an intensity level of potential demands in the trauma centers’ service coverage. As shown in Figure 1a, if the fatalities’ spatial pattern is fairly dispersed with similar values within the TDC, a lower statistic is computed (*G* = 0.0076) compared to the case of TDC’s clustered fatalities (Figure 1b, *G* = 0.0111). The *G*-statistic plays a role of weight for priority among trauma centers when they are selected via ACLP models. The TDC of candidate trauma centers with a higher *G* is chosen in preference to the lower ones. In terms of modeling perspective for the ACLP, the *G-*statistic generates a certain range of positive values (>=0), allowing the computed value to be integrated as weight for an integer decision variable {0, 1} for facility.

### 2.4. Locating Trauma Centers Using ACLP

The covering location problems focus on locating facilities geographically arranged to cover all or the maximal demands included within each facility’s standard of coverage. Depending on the goal, three models are worth considering: location set covering problem (LSCP), maximal covering location problem (MCLP), and anti-covering location problem (ACLP). The LSCP seeks to identify the minimum number of facilities to serve all the demand, consumers or populations, regardless of the costs of installation, maintenance expenditure, etc. [38]. In contrast, the MCLP maximizes the total coverage of demands given a limited number of facilities [44]. This model is used to explore the maximal possible service area with the allowable facility resources [45]. LSCP is applied to find better location of emergency medical service facilities to improve the performance of EMS [29] and lots of variants of the models are developed based on the MCLP models to find the optimal location for EMS and healthcare facilities [41,46,47,48,49].

The ACLP involves maximizing the number of selected facilities, but no two are placed within a separation standard. The ACLP has been used in numerous practical applications in diverse decision-making-related processes. Examples include homeland security [19], military defense location [50], forest harvest selection problems [51], location of undesirable facilities [52,53], locating criminal’s residency [54], potential store placement under separation standards [55], and planning commercial parcels [56]. However, up to our best knowledge, there was no attempt to apply the ACLP to find optimal EMS or trauma centers locations.

**Figure 2:**
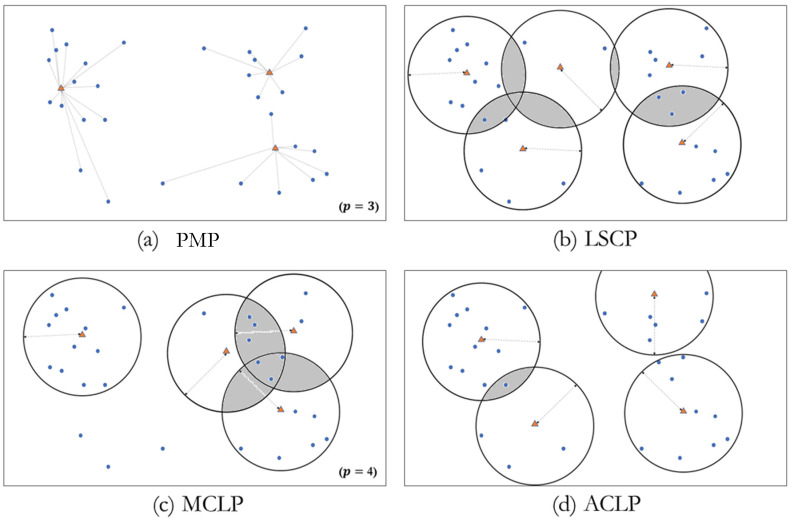
Difference of spatial arrangement of facility coverages among (**a**) PMP, (**b**) LSCP, (**c**) MCLP, and (**d**) ACLP.

In terms of model behaviors to cover demands, the three covering models can be compared with location-allocation problems, such as the *p*-median problem (PMP). Figure 2 shows the difference among four location problems. The PMP (Figure 2a) determines the location of facilities and the assignment of demands through a constraint for assignment between demand and facility. Using these characteristics, the PMP was applied to healthcare location problems (see [57]). In the context of medical service coverage, two underlying issues should be mentioned. First, the assignment is a point-to-point relationship, rather than an aerial coverage over demand location points. Second, any demand in the PMP should be allocated to one of the selected facilities; however, such an allocation is often infeasible in real emergencies, such as trauma incidents because a demand may not be covered by any EMS facility unless they are within the limited travel time or can be covered by multiple facilities. Patients too far from a facility do not have access to EMS.

One notable difference between the PMP and covering models (Figure 2b–d) is the possible creation of CSAs among facilities (shaded areas in Figure 2b–d). The PMP does not allow any CSAs because of the exclusive assignment condition between demands and facilities. In contrast, the LSCP model tends to increase the number of facilities until they cover all demands, it can entail many and large CSAs compared to the MCLP (Figure 2b). The MCLP is free from the assumption of complete coverage, but it may leave uncovered demands and allow excessive coverage for highly weighted demands (Figure 2c). ACLP also may have CSA; however, the number and size of CSAs created by ACLP are likely to be minimal compared to the outcomes of LSCP and MCLP due to minimum separation restriction among facilities (Figure 2d). In this context, compared to LSCP and MCLP, the ACLP is a different type of covering location problem dealing with equity implicitly rather than efficiency of the system because it maximizes the whole area of coverage while enhancing efficiency by minimizing the demands covered by multiple facilities.

For modeling a trauma center’s location, a CSA may be allowable in coverage models to some extent unless a CSA is covered by too many facilities, thus entailing other demands that may remain uncovered. The key difference between models for emergency medical facilities and for non-emergency facilities is how well the equity of service access is explored, manifested as a level of accessibility’s spatial disparity rather than the system’s efficiency (Ye and Kim 2016). The arrangement of the facilities’ coverage is geographically dispersed—thus minimizing the CSAs among facilities (Murray and Kim 2009), which is the rationale for why the ACLP can be employed to explore the solution to trauma centers’ location problem.

## 3. Data

### 3.1. Trauma Centers and General Hospitals in Tennessee

The locations of Tennessee’s healthcare facilities are available from Tennessee’s Department of Health [16]. Trauma centers are classified as one of three levels (I to III) depending on the treatments provided. As of 2019, Tennessee had 13 trauma centers (Level I: 5, Level II: 2, and Level III: 6). A general hospital can be designated as a trauma center if it meets the requirements of a facility, devices, and specifically qualified doctors who handle challenging emergency medical situations. The locations of general hospitals are geocoded based on the location information or mailing address from the Centers for Medicare and Medicaid Service (CMS) [58]. In this research, those general hospitals are considered candidates and are used when the ACLP models find the best locations for trauma centers.

**Figure 3:**
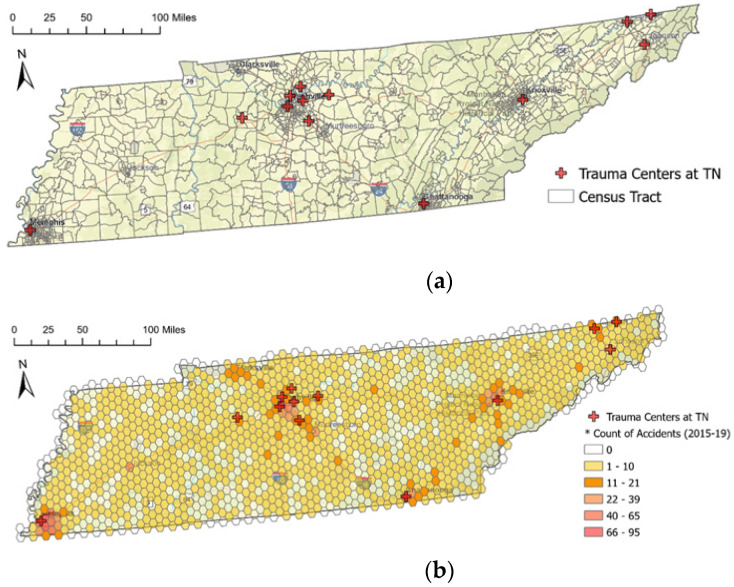
Study area of the state of Tennessee with the location of trauma centers, and data processing of hexagonal tessellation. (**a**) Locations of trauma centers and census tract in Tennessee, 2019; (**b**) Tessellation by hexagonal grids and vehicle accidents distribution.

Figure 3a portrays the study area and the locations of 13 trauma centers in Tennessee as of 2019. Among them, nine of the trauma centers are in urban areas; and the other four trauma centers are in rural areas near urban areas. Accessibility to trauma centers from the location of vehicle accidents in rural areas is inferior to accessibility to vehicle accidents in urban areas, indicating a strong presence of disparity in trauma services.

To explore the behaviors of the ACLP models determining the location of additional trauma centers in relation to existing centers, it is worth examining the number of general hospitals that are candidates for becoming trauma centers are accessible from a demand location. The number of facilities accessible from each demand location to the nearest trauma center in one hour is not enough to cover all the demands in need. In Tennessee, 80.35% of the population is under the one-hour coverage. However, the percentage of trauma center coverage is significantly higher for the urban population (88.31%) compared to the rural population (64.61%). Additionally, the potential demands in terms of the actual number of accidents (77.01%) and fatalities (76.57%) are far below the covered total population (80.35%). Yet, more than one fifth of the potential demands related to motor vehicle accidents are not accessible to the current trauma centers within an hour. However, accessibility to general hospitals is quite stable in all of the TDCs. If we include general hospitals as candidates for future trauma centers, the models can have an improved coverage percentage for the total population (100%), number of accidents (99.9%), and fatalities (100%), which enables the ACLP models to explore more feasible scenarios from a healthcare planning perspective.

### 3.2. Potential Demands

This study used three types of demands for the ACLP modeling: (a) population size, (b) number of vehicle accidents, and (c) number of fatalities. It is acknowledged that using population represents the potential demand because of data availability for specific spatial units from Census Bureau. The location of traumatic accidents is generally point data, indicating the existence of accident-prone areas. Note that the distribution of vehicle accidents does not follow the spatial distribution of population because most accidents occur along transportation networks. Additionally, the number of fatalities does not necessarily conform to the pattern of vehicle accidents. As such, the optimal location of trauma centers may vary with the variable defined as potential demand. Population data were obtained from the American Community Survey (ACS) for 2015 for a five-year estimation of census tract units [59]. According to the Census Bureau’s classification system, among 1497 census tracts, 66.1% (989) are urban and 33.9% (508) are rural in Tennessee. The Fatality Analysis Reporting System (FARS) provides details of car-related accidents and the resulting fatalities [60].

### 3.3. Tessellation of the Spatial Unit

The spatial unit of analysis was defined with the 25-square-mile area of hexagonal tessellation grids. All the demands’ information mentioned above was assigned to each hexagonal tessellation grid. Using hexagonal tessellation has two benefits: (1) enabling data synthetization from different sources and (2) minimizing spatial autocorrelation interference due to the features’ different locations. First, the demand data have different feature classes. Population data are represented at the areal unit (polygon), while vehicle accidents data are represented as points, requiring transforming one data representation to the other into the feature class’s uniformed format. Second, spatial autocorrelation can be affected by the data distribution depending on the distribution of points or polygons. For example, as displayed in Figure 3a, each census tract has a different shape and size. The census tracts in urban areas are much smaller than tracts in rural ones because the tracts are delineated by population size. Accident locations also tend to cluster at certain points of the region.

The hexagonal tessellation was useful to mash up two different types of spatial data (i.e., the polygon data for population and the points of accident locations) into a single unit standard. Hexagon tessellation was used because its shape is to alleviate the possibility of spatial autocorrelation by the different size of units by distributing the demands’ locations evenly with the unified shape and size of spatial units in the study area.

The census tract and FARS data overlaid each hexagonal unit to combine the population and vehicle accident information. The number of accidents and fatalities were aggregated into each grid. The spatial distribution of vehicle accidents is presented in Figure 3b.

### 3.4. Defining TDCs for Analysis

This research used the TDCs under different travel time conditions of 30, 45, 60, 75, and 90 min each. Patients in accidents located outside TDCs’ designated minutes cannot reach the facility within the defined time standard.

**Figure 4:**
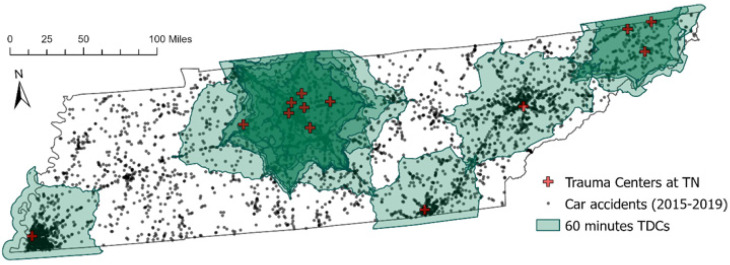
The 60 min TDCs from existing trauma centers and locations of vehicle accidents.

Figure 4 shows the location of vehicle accidents and the current 13 trauma centers in Tennessee with a 60 min TDC. Given the TDCs are depicted in Figure 4, most trauma centers exist inside or adjacent to urban areas. However, many accidents still occur outside the 60 min TDC in rural areas, making it hard to reach a trauma center within the golden hour. Additionally, as highlighted by the CSAs, the darker shaded areas with more than two TDCs, most of the TDCs overlap in urban areas, raising the issue of excessive supply in particular areas.

## 4. Methods

This research consisted of two lines of analyses. The first analysis assessed the accessibility to trauma centers in all the hexagonal grid’s centroids. The percentage of demands covered by the overall areas of TDCs was used in this analysis because it was intuitive to compare the beneficiaries according to different TDCs and model variations. The percentage of covered demand was calculated based on the sum of potential demands covered at least once out of the total demands. The potential demands in CSAs were also calculated in a similar manner; more than two facilities covered the demands. From a demand perspective, the number of accessible facilities was measured based on each demand location. The multiple numbers of facilities accessible from the demand locations are good for potential patients in the demand location. However, too many facilities accessible from the scarcer potential demands’ locations result in misusing resources.

**Figure 5:**
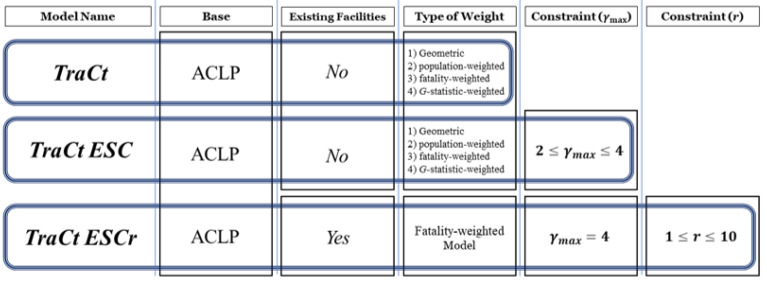
Structure of the modeling approach by different types of weight and constraints.

The second line of analysis involved the modeling approach to find the optimal location of trauma centers. Figure 5 illustrates the structure of this modeling approach. Three classes of ACLP for trauma center location problems were prescribed: (a) ACLP for a trauma center location problem (*TraCt*) as the base model, (b) ACLP for a trauma center location problem with excessive service control (*TraCt-ESC model*), and (c) ACLP for a trauma center location problem with excessive service control based on the planned number (***r***) of facilities (*TraCt-ESCr model*). First, the *TraCt* model assumes that no existing trauma centers are in the study area and that the number of new trauma centers is unrestricted. By applying various weights to the model, we explored to select the model covering the greatest number of potential demands with the least number of facilities chosen. Second, the *TraCt*-*ESC* models was prescribed to prevent excessive service supply to specific demand areas by applying an additional constraint to the *TraCt* model. The *TraCt*-*ESC* models share the assumption of the first model, and four different weights are also applied in this model. Third, considering the reality of the current trauma centers, a model was developed to establish additional facilities while maintaining the existing ones. This model is named the excessive service control model with restrictions maximum number of facilities available from each demand location (*TraCt*-*ESC*r). The additional constraints to the *TraCt*-*ESC*r model filter the facilities and demands that already exist and are covered, respectively.

### 4.1. TraCt Model

The standard ACLP *TraCt* models in this research were subject to the set of constraints (2) through (4). To model the ACLPs, a clique was prescribed as a constraint form (3) to meet the required separation among candidates or existing trauma centers (*Y_k_*). In the literature, there are three ways to prescribe cliques: pairwise, neighborhood, and hybrid [56]. In our ACLP models, a pairwise type of clique constraints was employed considering the computational expense for our input data:

(2) $$Maximize\;\underset{k}{\sum }\alpha_{k}Y_{k}$$

Subject to:

(3) $$Y_{k}+Y_{j}\;\leq 1\;\;\;\;\;\forall k,\;\;j\in \phi_{k}\;$$

(4) $$Y_{k}=\left\{0,1\right\}\;\;\;\;\;\forall k.$$

where *α_k_*: a weight representing the number of served demands when a candidate or existing trauma facility *Y_k_* is selected;

*Y_k_*: index of candidates or current trauma centers;

*Φ_k_*: the set of facilities *j* which is located inside of the facility *k’*s coverage.

The objective function (2) is to maximize the sum of weighted benefits covered by the TDCs of facilities *Y_k_*. Note that *α_k_* represents a weight, a metric of demand, which is covered by the TDC of *Y_k_*. TraCT has four standard models based on what *α_k_* is used: (a) geometric model (*α_k_* = 1), (b) population-weighted model (*α_k_* = size of population), (c) fatality-weighted model (*α_k_* = fatalities), and (d) *G-*statistic-fatality-weighted model (*α_k_* = *G-*statistic as a metric to show a level of intensity of clustering). The four standard models determine the location of facilities simply based on their TDCs. The model (d) calculates the *G-*statistic for the fatalities at TDC of *Y_k_* to represent *α_k_*. The constraints (3) force a facility *k* to keep separation with any candidate facilities *j* in the clique set *Φ_k_*. The constraints (4) impose a binary integer restriction on *Y_k_* (1 = if selected, 0 = otherwise).

### 4.2. TraCt-ESC Model

The models utilize the formulation of the standard model, (2) through (4) plus additional constraints (5). The maximum number of facilities covering each demand can be imposed by the constraints (5) to regulate the excessive supply for the specific demand locations. The constraints restricting excessive services (5) are identified below:

(5) $$\underset{j\in E_{i}}{\sum }Y_{j}\leq \gamma_{max}\;\;\;\;\;\;\;\;\;\;\;\forall i$$

where $\gamma_{max}$: the maximum number of facilities that can cover each demand location;

*E_i_*: the set of trauma facilities covering unit *i.*

The integer value *γ_max_* on the right hand in the constraints (5) forces the maximum number of trauma facilities allowed to serve the demand locations. In this research, four sets of incremental range *γ_max_* (=∞, 2, 3, 4) were used to examine the model behaviors to determine the trauma facilities’ locations.

### 4.3. TraCt-ESCr Model

The third model is for locating a limited number of additional facilities along with existing facilities. We called it the excessive service control model with restriction of maximum number of facilities (*r*) accessible from each demand location (*TraCt-ESCr*). The index *r* in the model represents the number of facilities to be added on the landscape of coverage by the existing trauma centers. The most distinctive aspect of the *TraCt-ESCr* model compared to prior models is that this model seeks to identify the optimal locations of additional *r* trauma centers considering predetermined coverage by the thirteen existing trauma centers. The *TraCt-ESCr* model is formulated via (6) to (10):

(6) $$Maximize\;\underset{t}{\sum }\alpha_{t}Y_{t}$$

Subject to:

(7) $$Y_{t}+Y_{u}\;\leq 1\;\;\;\;\;\forall t,\;\;u\in \phi_{t\;},$$

(8) $$\sum^{\;}Y_{t}=r$$

(9) $$\underset{u\in U_{i}}{\sum }Y_{u}\leq \gamma_{max}\;\;\;\;\;\;\;\;\;\;\;\;\;\forall i$$

(10) $$Y_{t}=\left\{0,\;1\right\}\;\;\;\;\;\;\;\;\;\;\;\forall t$$

where 

*α_t_*: a weight representing the number of fatalities when a candidate facility *Y_t_* is selected; 

*Y_t_*: candidates facilities excluding current trauma centers;

*Φ_t_*: the set of facility *u* that is located inside of the facility *Y_t_’*s coverage;

*r*: the number of facilities that can be established in addition to the existing trauma centers (1 ≤ *r ≤* 10);

*γ_max_*: the maximum number of facilities that is allowed to cover each demand location (*γ_max_* = 4 in this research);

*U_i_*: the set of candidate facilities covering demand location *i.*

The objective function (6) is to maximize the fatality-weighted benefits that the TDCs of facilities *Y_t_* cover. Constraint (7) stipulates the spatial separation among candidate facilities in a clique set *Φ_t_.* As noted, the *TraCt-ESCr* model excludes the existing trauma centers from candidates, and constraint (8) limits the number of *Y_t_*. Constraints (9) limit the maximum number of trauma facilities serving the demand location *i*. The setting of *γ_max_* = 4 indicates that the candidate facilities that cover demand location *i* with more than five trauma centers are not considered via these constraints. Constraint (10) imposes the binary integer restriction on the decision variables of *Y_t_*.

## 5. Results

### 5.1. Demands Covered by Trauma Centers in Tennessee

**Figure 6:**
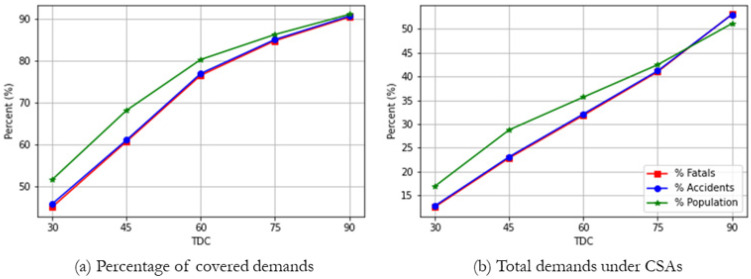
Potential demands covered by trauma centers in Tennessee, 2019. (**a**) Percentage of covered demands; (**b**) Total demands under CSAs.

As shown in Figure 6a, the percentage of fatalities, vehicle accidents, and the population within TDCs rises as the travel time distance of TDC increases from 30, 45, 60, 75, and 90 min. Only 46% of demands are covered within 30 min TDCs, but nearly 77% of the vehicle accidents and their fatalities are covered within 60 min TDCs from the trauma centers. Additionally, more than 90% of potential demands are covered by existing trauma centers within 90 min TDCs. As a reference, the coverage rate of population as demand is slightly greater than that of vehicle accidents, but no significant difference is observed. However, as implied in Figure 6a,b, of concern is that a significant portion of vehicle accidents (32.11%) occur in 60 min TDC’s CSAs. The rate of potential demands covered by CSAs increases with larger TDCs, indicating that the coverage of trauma centers is not well expanded to the uncovered areas; instead, the benefit is more established at CSAs, raising an issue of the system’s inefficiency of redundancy in providing service coverage equitably. In other words, many vehicle accidents still do not ensure the access within TDCs even with the expansion of coverage from TDCs of existing trauma centers. Given this fact, exploring the spatial arrangement of trauma centers with limiting excessive supply is necessary to see how to improve the service coverage for entire areas.

### 5.2. TraCt Model Solutions by Types of Weights

Four *TraCt* models are prescribed to explore the optimal location of trauma centers. Table 1 shows the number of facilities that need to cover entire Tennessee by (a) geometric model without weight, (b) population-weighted model, (c) fatality-weighted model, and (d) *G-*statistic-fatality-weighted model. As the travel time distance in TDC increases, the number of facilities decreases in all four models. Among them, the population-weighted model has the lowest number of facilities as a solution for 45, 60, and 75 min TDC. The fatality model has a moderate number of facilities in 60 min TDC, and the *G-*statistic-fatality-weighted model has the lowest number in 90 min TDC. Considering the 60 min TDC is important because it is the so-called “golden hour,” as the standard for emergency medical access, the population-weighted model seems to be the most effective model due to the smallest number of facilities needed to maximize demands covered by TDCs.

**Figure 7:**
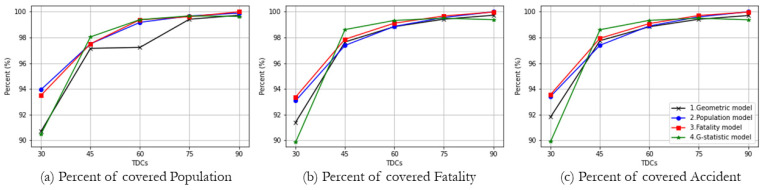
ACLP solutions from different weighted objectives by potential demands. (**a**) Percent of covered population; (**b**) Percent of covered fatality; (**c**) Percent of covered accident.

Figure 7 presents the results of the four *TraCt* models in terms of the percent of the total demands, such as populations, fatalities, and accidents covered by trauma centers with TDCs. The percent of demands covered by a solution is different because each model has differently weighted objectives. As a note, some models, especially when they performed with smaller TDCs such as 30 and 45 min, cannot serve all demands completely because the difference in locations of demands and candidates with small TDC result in inadequate coverage. All models show a pattern of the larger the TDC, the higher the percentage of demand covered. However, it is worthy to note that the geometric, population-weighted, and fatality-weighted models have an inflection point at which the increment stabilizes TDC of around 60 min, but the *G-*statistics model has an inflection point at a TDC of around 45 min. When a 60 min TDC is applied, emergency medical services can be provided for more than 98% of the demand in all *TraCt* models, indicating that 60 min TDC can work as the standard to determine the optimal location of a trauma center.

**Figure 8:**
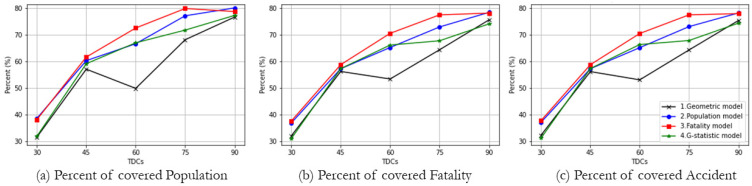
Potential demands under CSAs according to different objectives. (**a**) Percent of covered population; (**b**) Percent of covered fatality; (**c**) Percent of covered accident.

Figure 8 highlights that the potential demands covered in a CSA are worthy of investigation because the distribution of demands results in a different arrangement of trauma centers due to their CSAs, consequently revealing the different pattern in the percentage of service coverage shown in Figure 7. All models provide solutions by covering the same demand multiple times with different chosen facilities, CSA. The potential demands under CSA consequently increase the model objective, and all weighted models have a significant volume of demands covered by CSA.

As the overall pattern of figures (a), (b), and (c) is not much different, but the geometric model shows a unique pattern. Because the geometric model works with the number of demand locations, this model never takes care of the population, the number of accidents, and fatalities. The populations tend to locate in CSAs when we use population as an objective weight, and the fatality-weighted model has more demand than the other types of models. However, the *G-*statistic-fatality-weighted model has fewer potential demands within CSA regions. *G-*statistic has effects that choose the facilities that have fewer CSAs by focusing on the spatial distribution pattern of demands and facilities.

Figure 7 and Figure 8 together point that the *G-*statistic-fatality-weighted model has the highest percent of covered demands and the lowest percent of demands in CSAs, when the number of facilities is not limited. It reaches the inflection point at a 60 min TDC where a significantly high percentage of potential demands is covered. Specifically, while the number of demands covered by 75 min TDC is not substantially improved to those under the coverage of 60 min TDC, the covered demands under CSAs increase greatly from 60 min TDC to 75 min TDC. Thus, 60 min TDC has strength reduces the number of demands under CSAs, while keeping coverage of a similar percentage of demands.

**Figure 9:**
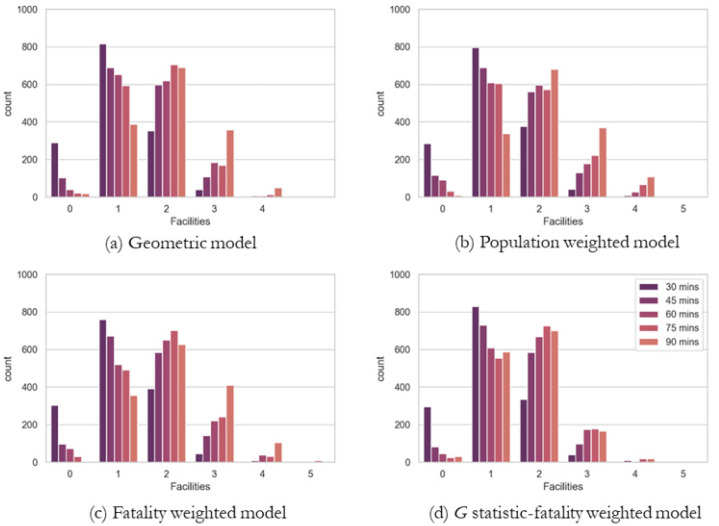
Number of facilities available from each demand by different *TraCt* models. (**a**) Geometric model; (**b**) Population weighted model; (**c**) Fatality weighted model; (**d**) *G* statistic-fatality weighted model.

Figure 9 shows the distribution of the number of facilities that are available for each demand location. As indicated by ‘0′ facilities in four models, a small TDC such as 30 min leave more than 300 demand locations uncovered, meaning no facilities are available within 30 min TDC. As TDCs are larger, more demands are covered with multiple facilities (see 2, 3, 4, 5 in the *x*-axis).

Although the patterns among four models are similar, there are two points to review. First, minimizing uncovered demands is critical. The demands with unavailability of trauma facilities are attenuated from 45 min TDC in all models and almost disappear in 90 min TDC. Second, too many trauma facilities available from demands imply a sign of excessive service for trauma cares, which can be translated as a system inefficiency in terms of emergency management planning. Furthermore, the population and fatality models are less efficient than the geometric model because the geometric model does not have the demands with five facilities accessible and barely has four facilities available from each demand. However, the solution of trauma facility locations by the *G-*statistic-fatality-weighted model indicates that more potential demands can access to trauma facilities at least once or twice, while minimizing the number of demands that are accessible from more than four facilities.

The analysis above presents the results of model solutions by measuring the percentage of fatalities, accidents, and population, and reviewed the exact number of facilities available for the demand locations to check the different levels of excessive service providing to some of the demands. Thus, the extended model exploring the model behaviors to minimize excessive trauma facilities by controlling the maximum number of facilities available for each demand location is necessary.

### 5.3. TraCt-ESC Model Solutions by Maximum Number of Accessible Facilities 

**Figure 10:**
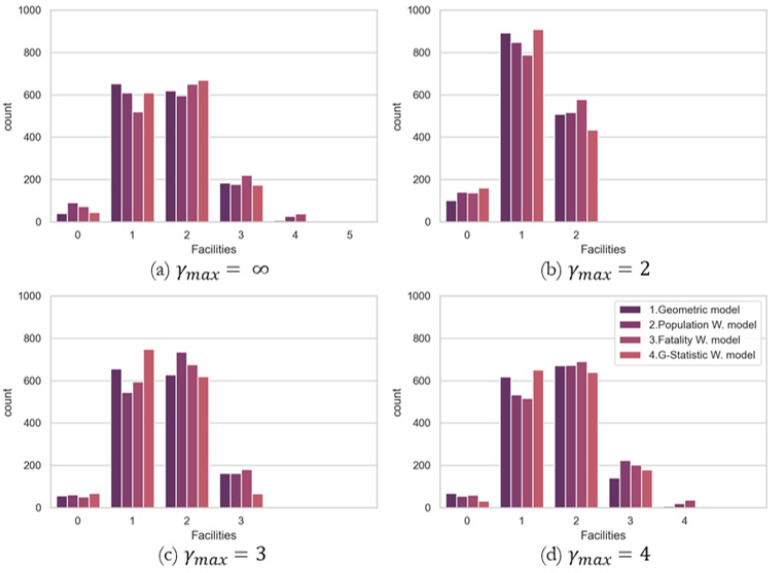
The number of facilities accessible from each demand location by the models and *γ_max_* for the *TraCt-ESC* model. (**a**) *γ_max_* = ∞; (**b**) *γ_max_* = 2; (**c**) *γ_max_* = 3; (**d**) *γ_max_* = 4.

Table 2 summarizes the results of the number of facilities chosen for each model to the range of *γ_max_*, and Figure 10 presents the number of facilities available from each demand when the 60 min TDCs is applied. For reference, the ‘$\gamma_{max}=\infty$’ in the first column of Table 2 is the same result of the *TraCt* model for 60 min TDC as there is no limitation for the multiple facilities accessible from each demand location. A lower number of *γ_max_* prevents the number of excessive service areas (CSAs) by restriction. However, the attenuation of CSAs does not necessarily entail the improvement of coverages that is measured as the maximum number of demands. The greater number of *γ_max_* allows a greater number of facilities and reduces the number of uncovered demands. 

Figure 10 presents the relationship between the number of facilities accessible from each demand location focusing on the different *γ_max_* (=∞, 2, 3, and 4) for 60 min TDC by four different weights. For reference, Figure 9 compares the TDC difference by model applications, but Figure 10 reviews the difference among the constraints *γ_max_*. A lower *γ_max_* needs a lower number of facilities, but leaves too many potential demands without accessible service facilities, especially for the *γ_max_* = 2 model. In contrast, the case of *γ_max_* = 4 has the least number of demands left without accessible trauma centers with 4–7 more facilities than the *TraCt-ESC* *γ_max_* = 2 model. There is a trade-off between minimizing uncovered demands and the number of facilities needed. There is no competition among models, but the user can choose the specific *γ_max_* according to their priority. If somebody wants to minimize uncovered demands, a higher *γ_max_* can work for it. On the other hand, if someone tries to control excessive service accessibility, they can choose the maximum number of accessible facilities they want to control. For this case study, both *γ_max_* of 3 and 4 are stable with a smaller number of demand locations without trauma facilities, but more demand locations have a chance to choose the trauma centers among two or three facilities.

It should be highlighted that the models by *TraCt* and *TraCt-ESC* are useful to identify the ideal locations of trauma centers with an assumption that the locations of candidate facilities (e.g., general hospitals) are qualified as trauma centers, and eligible to be the optimal solution. However, if the optimal arrangement of the trauma centers considering the existing trauma centers in Tennessee, thus, rectifying the distribution of trauma centers at once is impossible, the optimal solutions could provide more practical healthcare planning scenarios. The model with a practical approach is necessary to benefit underserved potential demands by providing additional facilities from the most urgent areas while maintaining the existing facilities.

### 5.4. TraCt-ESCr Model Solutions by Number of Planned Facilities along with Current Trauma Centers

An extra consideration in solving the *TraCt-ESCr* model is the currently operating trauma centers’ services in the model. In this case, demands not covered by facilities are only to be assigned to *r*-trauma candidate hospitals, and the trauma service facilities currently operating in Tennessee are not considered among the candidates to be assigned. The 60 min TDCs are applied to the additional models. Based on the results of *TraCt-ESC* models, the fatality-weighted model is used as a case study with the *γ_max_* = 4 using constraints (9). The large number of *γ_max_* is applied to minimize the uncovered demands and to maximize the opportunity to access service facilities in the *TraCt-ESCr* models.

As a note, the spatial cluster index of *G-*statistic-fatality-weighted model is not used in this application because the removal of some candidates and demands affects spatial distribution patterns, creating potential random interference to the original spatial distribution of candidates and demands used in the *TraCt* models. The random interference on the spatial distribution distorts the spatial autocorrelation, and the *G*-statistic could be biased to affect the model solutions and their implications.

**Figure 11:**
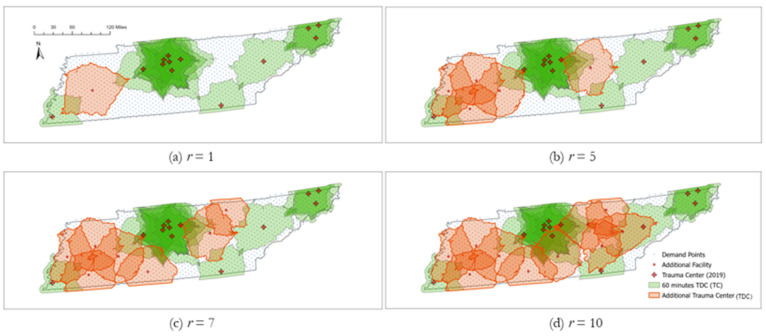
Locations of additional trauma centers according to the number of facilities available. (**a**) *r* = 1; (**b**) *r* = 5; (**c**) *r* = 7; (**d**) *r* = 10.

Figure 11 shows the model behavior of the *TraCt-ESC-r* with the range of *r* (=1 to 10), the number of planned new facilities, and the results for the selective *r* (=1, 3, 5, and 10) are displayed to examine the model behavior of how to respond expand the coverage with the additional trauma centers.

The noteworthy behaviors are summarized as follows. As illustrated in Figure 11a, establishing one additional trauma center (*r =* 1) results in the substantial improvement of coverage for unserved demands (fatalities: from 78.75 to 85.87%, accidents: from 79.09 to 86.07%, population: from 82.93 to 89.07%) and gradually cover the west area in Tennessee until *r* = 4. However, there is a little improvement the rate of coverage between r = 3 and 4, indicating that the coverage for the west area is saturated with *r* = 4. The number of demands covered by additional facilities grows again from *r* = 5 facilities and is stalled again in the range of 7 ≤ *r* ≤ 9, covering most of the Mideast Tennessee area (See Figure 11c). Finally, the 10th trauma center shown in Figure 1d begins to cover the south area, which is the most uncovered area at *r* = 9, resulting in the near complete coverage of the entire area with 97.77% of population, 96.69% of fatalities, and 95.77% of vehicle accidents, respectively.

## 6. Conclusions

In 2019, not all the patients involved in vehicle accidents in Tennessee had adequate access to trauma healthcare service within an hour’s travel time distance. Specifically, the lack of trauma care service for vehicle accidents was worse in some rural areas than in urban or suburban areas, as indicated in Figure 4. Many vehicle accidents even in rural areas could not receive timely treatment due to the distance to trauma centers.

The *TraCt* model finds the optimal location of trauma centers to minimize the uncovered location while pursuing efficiency by minimizing the number of facilities. The *TraCt* model finds better locations covering more beneficiaries with the smaller or same number of facilities to be established. This research demonstrates that the fatality and *G-*statistics-fatality-weighted *TraCt* model is advantageous for increasing the covered potential demands compared to the trauma centers’ current locations in the same limited minutes of TDCs. However, the *TraCt* models’ solutions do not fully guarantee the best efficiency. 

The *TraCt* model is designed to limit the maximum number of facilities while maintaining ACLP’s purpose, maximizing the number of beneficiaries. The additional constraints to providing the maximum number of facilities for each demand enhance the frugality of the model’s solutions. The *TraCt-ESC* model’s solution minimizes the demand points without accessible trauma centers and provides more options for selecting a trauma center from two to a predetermined number of facilities available in TDC.

Furthermore, the *TraCt-ESCr* model can be applied to assess additional facilities along with the existing trauma centers. This model is applicable at the state level by considering additional notations in addition to the existing facilities. The planned facilities can find the optimal location using the *TraCt-ESCr* model; the effect of this model’s application is noteworthy. Only one additional trauma center in rural areas raises the percentage of coverage dramatically. Some issues need to be explored in future research. The lack of information and/or data distortion is the most challenging problem, which is difficult to overcome. Many studies deal with these uncertainty issues and suggests improving the model structure or data processing [61]. Those approaches should be reviewed and applied to this model for better analysis and solutions.

In terms of the model design, the practical approach is required to optimize trauma centers’ locations along with the existing facilities. The empirical research shows that only one additional service can impact marginalized areas with trauma services more than a facility. Therefore, sequentially establishing additional facilities may be more efficient than the ‘r’ more facility model in this research.

Lastly, the spatio-temporal model approach is vital to find actual policy implications based on the evidence deployed in this paper. Establishing new trauma centers is difficult due to the tremendous upfront investment and the cost of facilities’ maintenance and human resources. The spatio-temporal model can provide clues to mitigate the problem of accessing trauma centers in underserved areas.

## Figures and Tables

**Table 1:** Number of facilities chosen as solutions by different objectives.

*TraCt* Model by the Type of Weight	TDCs				
	30 min	45 min	60 min	75 min	90 min
Geometric model	60	36	23	17	14
Population-weighted model	60	35	21	16	14
Fatality-weighted model	60	36	22	17	14
*G-*statistic-fatality-weighted model	60	36	23	17	12

**Table 2:** Number of facilities chosen as solutions for *TraCt-ESC* models with *γ_max_*.

ACLP TraCt-ESC Model *(60 min TDC)*	*γ_max_*			
	$\gamma_{max}=\infty$	$\gamma_{max}=2$	$\gamma_{max}=3$	$\gamma_{max}=4$
Geometric	23	19	22	23
Population-weighted model	21	16	22	23
Fatality-weighted model	22	17	21	22
*G-*statistic-fatality weighted model	23	18	21	23

## Data Availability

Data processed for this study can be provided on request.
